# Motion sickness whilst reading as a passenger in the car is highly predictive of vestibular migraine

**DOI:** 10.3389/fneur.2024.1426081

**Published:** 2024-08-14

**Authors:** Konstantina Rova, Lucia Joffily, Lara Carvalho, Elvira Cortese, Nehzat Koohi, Diego Kaski

**Affiliations:** ^1^Department of Clinical and Movement Neurosciences, Institute of Neurology, University College London, London, United Kingdom; ^2^ENT Department, General Hospital George Papanikolaou, Thessaloniki, Greece; ^3^Universidade Federal do Estado do Rio de Janeiro, Rio de Janeiro, RJ, Brazil; ^4^Department of Audiovestibular Medicine, Royal National ENT Hospital, London, United Kingdom; ^5^The Ear Institute, University College London, London, United Kingdom

**Keywords:** vestibular migraine, motion sensitivity, diagnostic biomarkers, dizziness, vestibular disorders, motion sickness

## Abstract

**Background:**

Vestibular Migraine (VM) is a prevalent vestibular disorder, affecting up to 2.7% of the general population. Despite the establishment of diagnostic criteria by the Bárány Society and its inclusion in the International Classification of Headache Disorders, the clinical diagnosis of VM remains challenging due to its complex pathophysiology and symptom overlap with other dizziness disorders. Motion sickness is a core feature of migraine and can be interrogated through simple questionnaires.

**Objective:**

This study aims to identify to what extent motion sensitivity can predict VM compared to other causes of dizziness.

**Methods:**

We conducted a cross-sectional study involving 113 patients from the vestibular neurology clinics at University College London Hospitals. Participants were categorized into VM, Persistent Postural Perceptual Dizziness (PPPD), combined VM and PPPD, and ‘other’ dizziness etiologies. Data on motion sickness history and dizziness during car travel were collected through structured interviews and analyzed using logistic regression to assess the predictive value of these symptoms for VM.

**Results:**

A substantial portion of patients with VM (91.2%) reported nausea or dizziness when reading as a passenger, a symptom significantly more prevalent than in those with PPPD or other dizziness diagnoses. Logistic regression indicated that VM patients are significantly more likely to experience these symptoms compared to non-VM patients, with an odds ratio suggesting a strong predictive value for this symptom in diagnosing VM.

**Conclusion:**

The findings highlight increased motion sensitivity while reading in a moving vehicle as a promising diagnostic tool for VM, offering a practical aid in clinical settings to distinguish VM from other vestibular disorders.

## Introduction

Vestibular Migraine (VM) is among the most prevalent vestibular disorders, affecting approximately 2.7% of the general population (1, 2). It is characterized by recurrent, spontaneous episodes of vertigo, ranging from 5 min to 72 h, often accompanied by migrainous features in at least half of the cases. Despite diagnostic criteria by the Committee for Classification of Vestibular Disorders of the Bárány Society in 2012 (3), as well as the inclusion of VM in the third edition of the International Classification of Headache Disorders (ICHD) (4), clinical diagnosis remains challenging. For example, some patients may only partially fulfill criteria for VM and it may therefore not be possible to make a timely and confident diagnosis (5). Moreover, differentiating VM from other episodic vertigo disorders, such as Menière’s disease, is not always straightforward, particularly in the early stages of either condition (6). Furthermore, persistent VM symptoms can evolve into a chronic vestibular disorder, such as Persistent Postural Perceptual Dizziness (PPPD) which can occur independently, or co-exist with the triggering diagnosis, further complicating the diagnostic and therapeutic process (7, 8).

The elusive nature of VM’s pathophysiology highlights the need for a predictive clinical biomarker to improve the accuracy and timely diagnosis. Motion sensitivity is well-established as a core feature of migraine (9–11) and perhaps more specifically VM (12) and is less commonly observed in peripheral vestibular disorders (11, 13). This heightened sensitivity is attributed to an abnormal visual and vestibular cortical interaction in VM (14, 15), with effects upon spatial orientation and multi-sensory integration (16). Previous work identified that probing motion sickness in the context of traveling in a vehicle (e.g., reading in the passenger seat of a car) is 4.3 times more likely in VM compared to Benign Paroxysmal Positional Vertigo (BPPV) (17), indicating that this symptom could be a reliable indicator for VM diagnosis.

Our study aims to establish the diagnostic value of sickness when reading in the passenger seat of a car. We explored motion sickness symptoms in a cohort including patients with VM, and those with other dizziness etiologies, to evaluate the potential of these symptoms as predictors for VM to aid bedside diagnosis. In addition, given the significant overlap between VM and PPPD symptomatology (8), we also grouped VM and PPPD patients to evaluate the prevalence of motion sensitivity in patients with combined pathologies. For other groups (e.g., BPPV), patients were excluded if there were co-existent features of VM.

## Methods

### Participants

This study was conducted on a cohort of 113 patients who sought outpatient consultation in vestibular neurology clinics at University College London Hospitals, UK. Consultations were recorded between the years 2023 and 2024 and included both in-person or telephone-based appointments.

### Group classification

Patients were classified into four diagnostic subgroups based on clinical evaluations by an experienced Neurologist (DK). Group allocation was as follows:

- VM Group: Thirty-four patients met the International Classification of Headache Disorders, 3rd Edition (ICHD-3) criteria for a diagnosis of vestibular migraine, or probable vestibular migraine (18).
- PPPD Group: Thirty patients were diagnosed with PPPD in accordance with the International Classification of Vestibular Disorders [ICVD, (19)].
- VM + PPPD Group: Fifteen patients had a combined diagnosis of VM and secondary PPPD.
- Other dizziness (OD) group: The remaining 34 patients were diagnosed with a range of established vestibular disorders including BPPV, Menière’s disease, Unilateral Vestibular Hypofunction, Bilateral Vestibular Hypofunction, Mal de débarquement syndrome, and hemodynamic orthostatic dizziness/vertigo, according to established diagnostic criteria (20).

### Motion sickness assessment

A telephone-based questionnaire was administrated to assess patients’ susceptibility to motion sickness. The questionnaire included the following items with a response option of yes, no, or sometimes (when applicable):

1. Have you ever suffered from travel or motion sickness? (yes/no)
2. Do you feel dizzy whilst driving? (yes/no)
3. Have you ever felt dizzy or nauseous while reading inside a car as a passenger? (yes/no).

## Statistical analysis

Responses were recorded and stored into an Excel spreadsheet. Statistical analyses were conducted using SPSSv28 software. We conducted Chi-square tests to analyze demographic and symptom frequency differences across patient groups, supplemented by one-way ANOVA for age variations with Tukey’s *post hoc* tests for pairwise comparisons. Where assumptions for the Chi-square test were not met, Fisher’s exact test was applied, especially for the symptom of dizziness while driving. Logistic regression analysis explored the predictive influence of VM and PPPD, including their interaction effects on symptoms such as dizziness or nausea when reading as a passenger. To enhance the reliability of our regression results, bootstrap methods were employed, providing a non-parametric way to assess variability and improve confidence in the estimated parameters.

## Results

### Demographic and baseline characteristics

A total of 113 patients participated in the study, consisting of 31 men and 82 women. A Chi- square test indicated significant differences in gender distribution across the groups (*p* < 0.001). Specifically, the proportion of females in the VM group (91%) was significantly higher compared to the OD group (47%). There were no significant gender differences noted between the other groups. The mean age of participants was 55 years. Descriptive statistics, including means and standard deviations, were calculated for age across four diagnostic groups (OD, VM, PPPD, VM + PPPD). Due to the non-normal distribution of age in the OD group and the potential for unequal variances, Welch’s ANOVA was used to compare mean age differences among the groups, showing significant age differences between the groups (*p* < 0.001), as expected (Table 1).

**Table 1:** Gender and age distributions across dizziness groups.

	OD (*n* = 34)	VM (*n* = 34)	PPPD (*n* = 30)	VM + PPPD (*n* = 15)	Total (*n* = 113)	*p*-value
Gender F/M (%)	16/18 (47.06%/52.94%)	31/3 (91.18%/8.82%)	22/8 (73.33%/26.67%)	13/2 (86.67%/13.33%)	82/31 (72.57%/27.43%)	<0.001^1^
Age (Mean ± SD)	63.91 ± 15.13	49.97 ± 16.54	56.83 ± 16.38	46.60 ± 10.52	55.54 ± 16.53	<0.001^2^

F, Female; M, Male; n, Number of participants; OD, Other Dizziness; VM, Vestibular Migraine; PPPD, Persistent Postural Perceptual Dizziness.

1 Chi Square Test.

2 Welch ANOVA.

### Clinical characteristics

#### Motion sickness history

**Figure 1:**
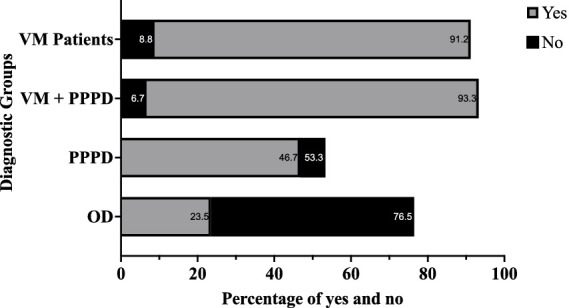
Bar chart showing the percentage of patients who reported feeling nauseous or dizzy when reading as passengers, compared to those who did not, across the four diagnostic groups. Blue bars represent patients reporting “Yes” to symptoms, while red bars indicate “No.” VM, Vestibular Migraine; PPPD, Persistent Postural Perceptual Dizziness; OD, Other Dizziness.

A substantial proportion of VM patients (*n* = 26, 76.5%) reported a history of motion sickness, significantly higher (*p* = 0.001) than those in other groups (*n* = 10, 66.7% of the VM + PPPD group, *n* = 11, 36.7% of the PPPD group, *n* = 12, 35.3% of the OD group; Figure 1). In pairwise comparisons, the VM group reported higher instances of motion sickness than both the OD and PPPD groups. No significant differences were found between the OD and PPPD groups in terms of motion sickness history.

### Dizziness while driving

Out of the patients who drive (85/113), 87.1% reported no dizziness while driving. Detailed analysis showed that the proportions of patients reporting dizziness while driving (*n* = 3, 10.3% of the VM group, *n* = 3, 23.1% of the VM + PPPD group, *n* = 3, 14.3% of the PPPD group, *n* = 2, 9.1% of the OD) did not differ significantly across the groups (*p* > 0.667).

### Dizziness while reading as a passenger

A significant 91.2% (*n* = 31) of the VM group and 93.3% (*n* = 14) of the VM + PPPD group reported feeling dizzy or nauseous while reading in a car, significantly higher than the PPPD (*n* = 14, 46.7%) and OD groups (*n* = 8, 23.5%) (*p* < 0.001). The difference between VM and VM + PPPD was not statistically significant, nor was it between the PPPD and OD groups (*p* > 0.1).

### Logistic regression

Logistic regression analysis was conducted to determine the effects of VM, PPPD, and their interaction (VMxPPPD) on the likelihood of experiencing dizziness or nausea while reading as a passenger. The analysis included 79 participants after applying inclusion criteria to filter the relevant cases. The regression model was statistically significant [*χ*^2^(2) = 20.297, *p* < 0.001], explaining between 22.7% (Cox & Snell R square) and 33.4% (Nagelkerke R Square) of the variance, and correctly classified 77.2% of cases.

Significant predictors included VM with an odds ratio [Exp(B)] of 16.0 (95% CI [1.860, 137.608], *p* = 0.012), indicating that participants with VM were significantly more likely to experience dizziness or nausea compared to those without these conditions. PPPD did not significantly predict the outcome (Exp(B) = 1.355, 95% CI [0.129, 14.199], *p* = 0.800).

Bootstrap results, based on 1,000 samples, confirmed the robustness of these findings, particularly for VM. Bootstrap bias and standard error indicated stable estimations across resampled datasets, enhancing the reliability of our estimates and providing a non-parametric way to assess variability and confidence in the estimated parameters.

### Sensitivity and specificity

The analysis of sensitivity and specificity was performed by dividing the groups into “total VM” (VM plus VM + PPPD) and “without VM” (OD plus PPPD). For the question on travel or motion sickness, the sensitivity for detecting VM was 73.5%, and the specificity was 64.1%. Dizziness while driving had a sensitivity of 14.3% and a specificity of 88.4%. The sensitivity for reporting dizziness or nausea while reading as a passenger was notably high at 98.1%, with a specificity of 65.6%.

## Discussion

Motion sickness is a feeling of unwellness caused by motion, especially during traveling and virtual reality immersion, leading to autonomic responses (nausea, vomiting, pallor, sweating, hypersalivation, and stomach awareness) (9, 10). Vestibular, somatosensory, and visual afferents inform our brain about body posture and movements. The concept of sensory conflict remains the most current explanation for motion sickness. Such conflicts arise when information from different sensory systems contradict expectations (12, 21). Previous studies have suggested a high correlation between motion sickness and VM (17, 22, 23). However, none of the previous studies systematically evaluated which specific question to ask a patient as a means of screening for VM.

Our findings are consistent with those of Patel and colleagues (17), who assessed the impact of the head shaking on vestibulo-ocular gain in patients with VM but also explored whether their participants experienced dizziness when reading as passengers in a car. Their results showed that all VM patients reported dizziness, compared to only half of those with BPPV, and none of the control group. In the current study, we did not specifically assess motion sickness in patients with migraine, in the absence of dizziness. However, Akdal et al. (22), recently concluded that migraine patients, regardless of a vertigo history, were significantly more prone to motion sickness and find reading as passengers more challenging than healthy individuals do. This suggests that motion sensitivity may be a general feature of migraine.

**Figure 2:**
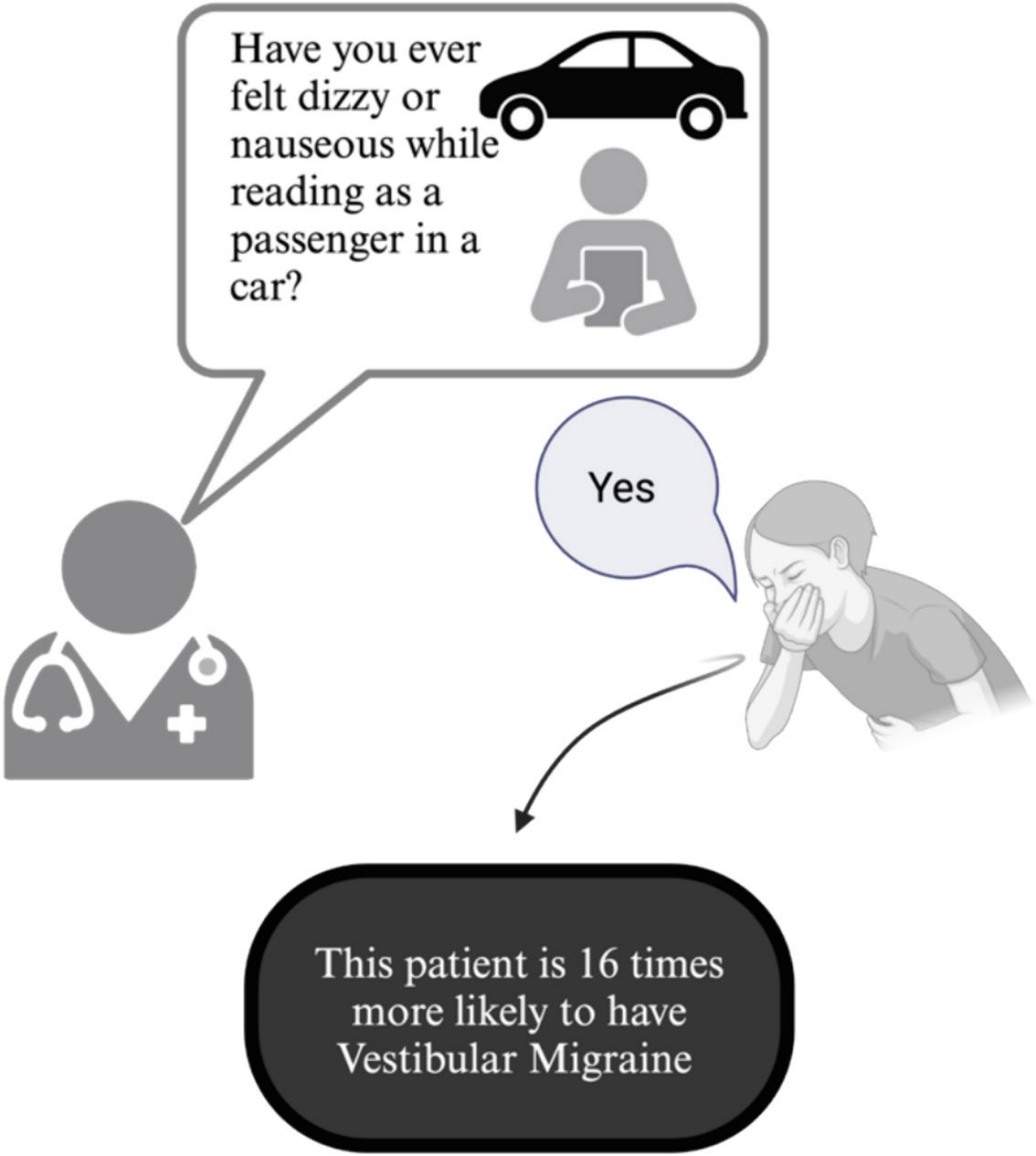
Clinical diagnostic utility of asking patients with episodic vertigo whether reading while in a passenger seat of a moving car induces dizziness or vertigo to identify vestibular migraine.

In our study, 76% of VM patients reported travel or motion sickness (irrespective of reading). This was significantly higher than other patients with dizziness, including those with PPPD. However, when specifically asked about feelings of nausea or dizziness *while reading as a passenger*, this rate increased to 91% among VM patients. Our logistic regression analysis revealed that the high number of responders with dizziness/nausea when reading in a passenger seat of a car in the VM + PPPD group is driven by the presence of VM. Thus, in a clinical setting, a positive response to this question corresponds to a 92% likelihood of encountering a patient suffering from VM, whether isolated or in conjunction with any other dizziness cause. This result confirms that this simple question can be used as an effective screening tool during history taking of a dizzy patient, given its high sensitivity (Figure 2) — a positive response is 16 times more likely in VM patients, providing a valuable diagnostic aid in ambiguous cases.

Other studies have used questionnaires, such as Motion Sickness Susceptibility Questionnaire (MSSQ) to assess motion sickness in patients with VM. However, such studies demonstrate no clear association between MSSQ scores and definite VM criteria. For example, in a study of undergraduate students with self-perceived motion sickness, 50% were found to meet criteria for VM, suggesting a predisposition to VM in those with high MSSQ scores (24). Others have demonstrated the reverse relationship where VM patients have elevated motion sickness and MSSQ scores compared to controls (24). From a pragmatic perspective, administering a full 40-item questionnaire is impractical in a clinical setting. Our data suggest it may be more efficient and effective to ask a single focused question, but that it must specifically link reading and motion (visuo-vestibular), not motion (vestibular) alone. We also wish to highlight that the presence of motion sickness when reading in a moving vehicle is not diagnostic of VM across a general population—its presence may simply denote a higher susceptibility relative to VM than an individual who has no problem reading in a moving vehicle.

It is interesting that individuals with travel sickness rarely suffer from motion sickness as drivers, a finding that we can now extend to patients with dizziness, irrespective of the cause—87% of patients reported no dizziness while driving. Patients with PPPD manifest a heightened awareness of self-motion and environment motion (25) leading to persistent dizziness but may paradoxically find relief from their persistent dizziness while driving. On the one hand, this may be because they can control and anticipate their body’s movement in response to vehicle motion (26). Indeed, a key feature of PPPD is a partial loss of agency over the body that underpinned by predictive coding models (27) so driving may facilitate more accurate predictions of self-motion. On the other hand, driving may distract them from focusing excessively from their bodily sensations.

Our study has notable limitations. Firstly, motion sickness is more common in women (28). Given that VM is far more common in women than in men, it is difficult to disentangle effects of gender on motion sickness in VM. Indeed, our groups were not matched for gender or age, reflecting the expected distribution on these for each condition (e.g., VM is more prevalent in younger females whereas in other vestibular conditions age and gender may be more evenly distributed). Secondly, we are unable to comment on the predictive value of our questions for diagnosis of VM in a non-dizzy population so our data can only be extrapolated to a clinical setting of patients with dizziness.

## Conclusion

Our study reinforces the significance of symptomatic evaluation in diagnosing VM, particularly the symptom of dizziness while reading as a passenger in a vehicle. With 91% of VM patients reporting this symptom, this question seems to be a highly sensitive diagnostic indicator in patients with dizziness. The distinct prevalence of this symptom among VM patients compared to those with other dizziness disorders, including PPPD, emphasizes its potential as a reliable screening tool in clinical assessments.

## Data availability statement

The original contributions presented in the study are included in the article/supplementary material, further inquiries can be directed to the corresponding author.

## Ethics statement

Ethical approval was not required for the study involving humans in accordance with the local legislation and institutional requirements. Written informed consent to participate in this study was not required from the participants or the participants’ legal guardians/next of kin in accordance with the national legislation and the institutional requirements.

## Author contributions

KR: Data curation, Methodology, Project administration, Resources, Writing – original draft, Writing – review & editing. LJ: Data curation, Formal analysis, Investigation, Methodology, Validation, Visualization, Writing – original draft, Writing – review & editing. LC: Data curation, Formal analysis, Writing – original draft, Writing – review & editing. EC: Data curation, Methodology, Visualization, Writing – original draft. NK: Data curation, Formal analysis, Investigation, Methodology, Project administration, Supervision, Validation, Visualization, Writing – original draft, Writing – review & editing. DK: Conceptualization, Data curation, Formal analysis, Investigation, Supervision, Visualization, Writing – original draft, Writing – review & editing.
